# Longitudinal associations between social anxiety symptoms and cannabis use throughout adolescence: the role of peer involvement

**DOI:** 10.1007/s00787-015-0747-8

**Published:** 2015-08-08

**Authors:** Stefanie A. Nelemans, William W. Hale, Quinten A. W. Raaijmakers, Susan J. T. Branje, Pol A. C. van Lier, Wim H. J. Meeus

**Affiliations:** Research Centre Adolescent Development, Utrecht University, PO Box 80.140, 3508 TC Utrecht, The Netherlands; Department of Developmental Psychology, VU University Amsterdam, Amsterdam, The Netherlands; Department of Developmental Psychology, Tilburg University, Tilburg, The Netherlands

**Keywords:** Adolescence, Social Anxiety Disorder (SAD) symptoms, Cannabis use, Longitudinal, Developmental psychopathology

## Abstract

There appear to be contradicting theories and empirical findings on the association between adolescent Social Anxiety Disorder (SAD) symptoms and cannabis use, suggesting potential *risk* as well as *protective* pathways. The aim of this six-year longitudinal study was to further examine associations between SAD symptoms and cannabis use over time in adolescents from the general population, specifically focusing on the potential role that adolescents’ involvement with their peers may have in these associations. Participants were 497 Dutch adolescents (57 % boys; *M*_age_ = 13.03 at T_1_), who completed annual self-report questionnaires for 6 successive years. Cross-lagged panel analysis suggested that adolescent SAD symptoms were associated with less peer involvement 1 year later. Less adolescent peer involvement was in turn associated with lower probabilities of cannabis use as well as lower frequency of cannabis use 1 year later. Most importantly, results suggested significant longitudinal indirect paths from adolescent SAD symptoms to cannabis use via adolescents’ peer involvement. Overall, these results provide support for a protective function of SAD symptoms in association with cannabis use in adolescents from the general population. This association is partially explained by less peer involvement (suggesting increased social isolation) for those adolescents with higher levels of SAD symptoms. Future research should aim to gain more insight into the exact nature of the relationship between anxiety and cannabis use in adolescents from the general population, especially regarding potential risk and protective processes that may explain this relationship.

## Introduction

Social Anxiety Disorder (SAD) symptoms are one of the most prevalent manifestations of psychopathology during adolescence [1] and appear to be related to a wide range of negative developmental outcomes and psychosocial problems in adolescence and adulthood [2–4]. Adolescence also goes together with a sharp increase in drug use, particularly cannabis use [5]. Substance use appears to be socially embedded during adolescence (i.e., closely related to adolescents’ involvement with peers; [6, 7]) and some experimentation with cannabis may be considered normative for this developmental period. At the same time, cannabis is generally an adolescent’s first contact with illicit drugs and is believed to be an important precursor of other illicit drug use (i.e., the gateway-hypothesis; [8]) and drug-related problems [9, 10]. Given that cannabis is the most widely used illicit drug in adolescence [11, 12], SAD symptoms are among the most prevalent forms of adolescent psychopathology, and both have been related to a wide range of negative developmental outcomes, research on developmental processes underlying associations between SAD symptoms and cannabis use during adolescence is of great interest to researchers and clinicians alike.

There appear to be contradicting theories and empirical findings regarding associations between SAD and cannabis use. While a specific relationship between SAD (vs. other anxiety symptoms) and cannabis use has been suggested [13], theory and empirical findings have suggested both positive associations (i.e., *risk* processes) and negative associations (i.e., *protective* processes). Theoretically, self-medication is the most commonly proposed risk process linking SAD symptoms and cannabis use. According to the “self-medication hypothesis”, adolescents with high levels of SAD symptoms are motivated to use cannabis to cope with and alleviate their symptoms [14] or because they anticipate that being under the influence of cannabis will make it easier to interact with peers. This hypothesis fits within the broader network of motivational [15] and tension-reduction models [16]. In contrast, according to the “buffer perspective” the social impairment associated with SAD symptoms likely limits adolescents’ contact with peers and may thereby limit both the availability of cannabis itself and exposure to peer influences or potential modeling of peer behavior [7, 17]. In addition, high levels of fearful traits that are central to SAD symptoms may serve a protective function as they go together with overcontrolling tendencies and fear of engaging in (health) risk behaviors such as cannabis use [18].

Empirically, support for both aforementioned theories has been found. The bulk of research appears to be in line with the self-medication hypothesis and suggests positive associations between SAD symptoms and cannabis use. These studies are characterized by psychiatric comorbidity rates in epidemiological samples [19, 20], studies that involve current cannabis users only [21], studies that focus on cannabis use problems and dependence rather than use in itself [22], and studies with undergraduate or adult samples (see [23] for a comparable discussion on associations between SAD symptoms and alcohol use). In contrast, results from the few existing cross-sectional [24] and longitudinal [25, 26] studies in adolescent community samples that include dimensional measures of SAD and cannabis use appear to be in line with the buffer perspective. Specifically, these studies suggest negative associations between SAD symptoms and cannabis use. This line of research in adolescents from the general population has, however, received much less attention. Hence, there is a clear need to further examine associations between SAD symptoms and cannabis use in adolescent community samples to better understand developmental processes that occur before SAD symptoms and cannabis use have reached problematic or clinical levels. This requires prospective longitudinal designs starting in early adolescence (before exposure to cannabis generally takes place) with multiple assessments until late adolescence to examine how associations between SAD symptoms and cannabis use evolve during this developmental period.

Moreover, there has been limited attention to how general processes of adolescence and developmental factors (e.g., increased focus on peers and social rewards) affect adolescent substance use patterns that may be related to later substance abuse and addiction. In light of the aforementioned theories that emphasize the social context of adolescent cannabis use, adolescents’ involvement in peer contexts may play an important role in associations between adolescent SAD symptoms and cannabis use. Symptoms of SAD are strongly associated with social impairment and negative peer outcomes [2, 3, 27] and likely disrupt adolescents’ normatively increasing involvement with peers [28]. Generally speaking, peer interactions and relationships have great potential to contribute to positive development in adolescence (e.g., academic achievement, school adjustment, identity formation, and autonomy development) as well as adulthood (e.g., social functioning, mental health, and health-related quality of life) [28]. The peer context affords opportunities for adolescents to explore boundaries, to learn about themselves and the world outside of the family context, and to practice social skills necessary for the establishment and maintenance of relationships with age-similar peers, as well as acquire other behavioral and emotional competencies [28–32]. However, adolescents’ involvement with their peers is also a well-established risk factor for adolescent cannabis use [33, 34].

Therefore, in the present study, we aimed to examine how adolescent SAD symptoms are associated with cannabis use in a community sample of adolescents followed from early to late adolescence. We thereby specifically focused on the potential role of adolescents’ involvement with peers in these longitudinal associations. In line with the buffer perspective, we expected higher SAD symptoms to be associated with less peer involvement, and less peer involvement to be associated with less cannabis use. Most importantly, we expected that adolescents’ peer involvement would partially explain longitudinal associations between adolescent SAD symptoms and cannabis use (i.e., statistically significant indirect effects). Compared to the categorical approaches most often used in previous studies, dimensional approaches have been suggested to provide a more reliable and valid assessment to measuring psychopathology [35] and to be particularly useful in community samples. Furthermore, this study adds to the current literature using an advanced statistical approach (i.e., Zero-Inflated Poisson model) to accurately capture the distribution of cannabis use in adolescents from the general population. Finally, this study is one of the first to examine potential processes underlying associations between adolescent SAD symptoms and cannabis use by focusing on the role of adolescent peer involvement in these associations. This study may thereby contribute to the understanding of how different developmental changes in adolescence are interrelated over time, as well as the potential processes underlying associations between adolescent SAD symptoms and cannabis use.

## Method

### Participants

Participants were 497 Dutch adolescents (57 % boys), with a mean age of 13.03 years (*SD* = 0.46) at the start of the study. Data for this study are part of the ongoing Research on Adolescent Development And Relationships (RADAR) Young project. All participants attended the first grade of secondary school at the start of the study. Family SES, based on parents’ job level, was low for 10.8 % of the participants. Sample attrition in the first six waves of RADAR Young was low across waves, with 425 of the 497 adolescents still participating at the sixth wave (i.e., cumulative retention rate of 85.5 %). Adolescents participating at all waves were slightly younger than those dropping out of the study, *p*(1, 495) = 6.61, *p* = 0.01, *η*^2^ = 0.01, but there were no significant differences in gender, *χ*^*2*^(1) = 0.60, *p* = 0.44, or SAD symptoms, peer involvement, and cannabis use at the start of the study, *F*(3, 464) = 0.29, *p* = 0.83. Little’s Missing Completely at Random test showed a normed *χ*^*2*^ (*χ*^2^/df) of 1.50, suggesting good fit between sample scores with and without imputation [36]. Missing data were handled in M*plus* by Full Information Maximum Likelihood [37].

### Procedure

Participants were recruited from randomly selected schools in the western and central regions of the Netherlands. Before the start of the study, participants and their parents received a complete description of the study and 70 % of the selected families provided active written informed consent (*N* = 497). The final RADAR sample contained only one adolescent (with some rare exceptions including two adolescents) from every school. Each year for 6 successive years, adolescents completed annual self-report questionnaires during a home visit. Participants received a small monetary compensation for every wave they completed the questionnaires. This study has been approved by the board of the local research institute and by the Medical Ethical Committee of the Utrecht Medical Centre, the Netherlands.

### Measures

#### SAD symptoms

We used the 4-item SAD subscale of the Dutch version of the original 38-item Screen for Child Anxiety Related Emotional Disorders (SCARED; [38, 39] ) to assess adolescent SAD symptoms. Adolescents rated the items on a 3-point scale, ranging from 1 (*almost never*) to 3 (*often*). A sample item includes “I feel nervous with people I don’t know well”. In this study, reliability of the SAD subscale was good over all waves (α = 0.78–0.86). Psychometric properties of the SCARED have shown to be good in previous studies [38–40].

#### Peer involvement

We assessed adolescents’ peer involvement by asking participants about their time spent with peers on weekdays and in the weekend with the 5-item “intensity of contact with friends” subscale of the Questionnaire on Peer Relationships [41]. Adolescents rated the items on a 3-point scale, ranging from 1 (indicating low peer involvement) to 3 (indicating high peer involvement). Sample items included “How much time do you spend with your peers on weekdays after school time?” and “How often do you meet with your peers in the weekend (Saturday and Sunday)?”. In this study, reliability of the peer involvement subscale was acceptable over all waves (α = 0.66–0.72).

#### Cannabis use

One item was used to assess adolescents’ cannabis use: “In the past 12 months, how often have you used weed, marihuana or hashish?”. Adolescents rated the item on a 14-point scale, ranging from 0 (*zero times*) to 10 (*10 times)*, followed by *11 to 19 times*, *20 to 39 times*, and *40 times or more*.

### Statistical analysis

We constructed a six-wave longitudinal cross-lagged panel model of adolescent SAD symptoms, peer involvement, and cannabis use in M*plus* 7.2 [37].^1^ Maximum likelihood estimation with standard errors and Chi-square robust to non-normality was used (MLR estimator). Since the distribution of cannabis use was highly positively skewed with many adolescents reporting zero-use of cannabis in the past 12 months (especially in early adolescence, the first years of our study), we used a Zero-Inflated Poisson (ZIP; [42, 43]) model to accurately capture this distribution. This type of modeling is appropriate for variables involving infrequent events, such as adolescent cannabis use in the general population, and conceptualizes the occurrence (yes/no) and frequency of cannabis use as separate but interrelated aspects that are estimated by separate equations within one statistical model. Thereby, we were able to examine how adolescent SAD symptoms and peer involvement were related to the probability of using cannabis (yes/no; referred to as “cannabis non-use”) as well as the frequency of use for cannabis users (referred to as “cannabis frequency”).

Our baseline model was a full ZIP mediation model, in which longitudinal parameters were constrained to be time invariant for reasons of parsimony. Specifically, we included one-year autoregressive paths for SAD symptoms, peer involvement, and cannabis frequency, which represent stability coefficients of each of these constructs over a one-year period. We further included concurrent associations between SAD symptoms and peer involvement over all 6 years, which represent associations between these constructs within each year over all 6 years. Most important to our research aim, we included one-year cross-lagged paths from SAD symptoms to peer involvement and from peer involvement to cannabis non-use and cannabis frequency, as well as all possible reverse cross-lagged paths, which represent regression paths from one constructs to another construct 1 year later (e.g., SAD symptoms at T_1_ predicting peer involvement at T_2_). Finally, we also included the two-year direct cross-lagged paths from SAD symptoms to cannabis non-use and cannabis frequency, as well as all possible reverse cross-lagged paths, which represent regression paths from one constructs to another construct 2 years later (e.g., SAD symptoms at T_1_ predicting cannabis use at T_3_). Sex was included as a covariate.

Because fit indices such as CFI and RMSEA are not available for ZIP models, we compared our baseline model to more complex models (i.e., where certain cross-paths were freely estimated over time) and more parsimonious models (i.e., where certain cross-paths were trimmed from the model) based on differences in sample-size adjusted (SSA) BIC and Satorra-Bentler scaled *χ*^2^-difference tests (i.e., Δ${{\chi }}_{\text{SB}}^{ 2}$; [44]) to get an idea of relative fit to alternative models. In addition to the unstandardized coefficients (*b*) that are provided by M*plus*, we calculated standardized coefficients (*β*) for the linear part of our cross-lagged model as well as the incidence rate ratios (*IRR*) or odds ratios (*OR*) for the ZIP part of our model to facilitate interpretation of the coefficients. All estimations of the longitudinal indirect effects were based on M*plus* estimation of indirect effects [37].^2^

## Results

### Descriptive statistics

Table 1 provides an overview of means and standard deviations of all study variables over 6 years. Rank-order stability between successive waves was moderate to high for all study variables, with correlations ranging between 0.50 and 0.72 for SAD symptoms, between 0.54 and 0.60 for peer involvement, and between 0.42 and 0.80 for cannabis use. Furthermore, within-wave correlations between all study variables were relatively modest and ranged between −0.13 and −0.20 for SAD symptoms and peer involvement, between 0.13 and 0.27 for peer involvement and cannabis use, and between −0.00 and −0.09 for SAD symptoms and cannabis use.^3^

**Table 1:** Descriptive statistics of all study variables

Variable	Age 13 (T_1_)		Age 14 (T_2_)		Age 15 (T_3_)		Age 16 (T_4_)		Age 17 (T_5_)		Age 18 (T_6_)	
	*M*	*SD*	*M*	*SD*	*M*	*SD*	*M*	*SD*	*M*	*SD*	*M*	*SD*
Social anxiety symptoms	1.64	0.51	1.46	0.50	1.48	0.53	1.45	0.50	1.43	0.50	1.42	0.51
Peer involvement	2.02	0.41	2.06	0.39	2.13	0.39	2.18	0.39	2.21	0.35	2.18	0.38
Cannabis use frequency	0.09	0.83	0.23	1.36	0.79	2.47	1.39	3.33	2.06	4.07	2.53	4.38

Social anxiety symptoms and peer involvement ranged from 1 to 3. Cannabis use ranged from 0 to 13 (counts)

In line with previous suggestions [5], cannabis use appeared to be nearly absent in early adolescence but steeply increased throughout adolescence. Specifically, at the start of our study (when adolescents were approximately 13 years old) only 2 % of adolescents reported having used cannabis at least once in the past 12 months, whereas by the end of our study (when adolescents were approximately 18 years old) 37 % of adolescents reported having used cannabis at least once in the past 12 months. Adolescents’ cannabis frequency also increased over time. At the start of our study, approximately 50 % of adolescent cannabis users reported having used cannabis only once in the past 12 months, whereas by the end of our study 84 % of adolescent cannabis users reported having used cannabis more than once in the past 12 months.

### Adolescent SAD symptoms, peer involvement, and cannabis use

For reasons of parsimony, we constrained all longitudinal parameters in our baseline cross-lagged panel model to be time invariant, SSA BIC = 9581.78. Freeing all parameters of interest (i.e., all cross-lagged paths) in our model did not significantly improve the model fit, SSA BIC = 9640.38, Δ${{\chi }}_{\text{SB}}^{ 2}$(29) = 19.73, *p* = 0.90, ΔSSA BIC = 58.61, so we kept all parameters in our model constrained to be time invariant for reasons of parsimony [45]. Trimming the insignificant direct cross-lagged paths between adolescent SAD symptoms and cannabis use from our model also did not result in a significantly worse model fit, SSA BIC = 9580.84, Δ${{\chi }}_{\text{SB}}^{ 2}$(3) = 5.61, *p* = 0.13, ΔSSA BIC = 0.94, so we retained the more parsimonious trimmed model.

**Fig. 1:**
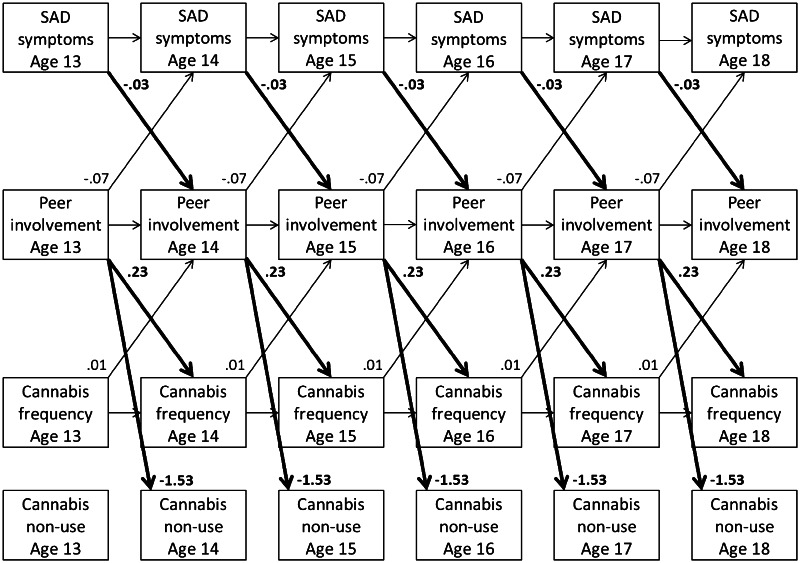
SAD = Social Anxiety Disorder. Unstandardized longitudinal associations between adolescent SAD symptoms, adolescent peer involvement, and adolescent cannabis non-use and frequency of cannabis use over time. Although not displayed for reasons of clarity, this model also includes concurrent associations between SAD symptoms and peer involvement and sex as a covariate

In this final cross-lagged panel model (see Fig. 1), girls reported somewhat higher levels of SAD symptoms over time, *b* = 0.10, 95 % CI = [0.064, 0.133], *β* = 0.19‒0.20, *p* < 0.001, a higher probability of cannabis non-use, *b* = 0.61, *OR* = 1.84, 95 % CI_*OR*_ = [1.300, 2.599], *p* = 0.001, and lower cannabis frequency, *b* = −0.19, *IRR* = 0.83, 95 % CI_*IRR*_ = [0.721, 0.957], *p* = 0.01, compared to boys, but there were no significant differences in peer involvement, *b* = −0.02, 95 % CI = [−0.045, 0.007], *p* = 0.16.^4^ Other paths in this model that were not directly linked to our research aim included one-year autoregressive paths for SAD symptoms, *b* = 0.61, 95 % CI = [0.561, 0.658], *β* = 0.59‒0.64, *p* < 0.001, peer involvement, *b* = 0.55, 95 % CI = [0.503, 0.598], *β* = 0.52‒0.61, *p* < 0.001, and cannabis frequency, *b* = 0.08, *IRR* = 1.08, 95 % CI_*IRR*_ = [1.067, 1.093], *p* < 0.001, over all 6 years, as well as concurrent associations between SAD symptoms and peer involvement at T_1_, *cov* = −0.04, 95 % CI = [−0.057, −0.019], *r* = −0.18, *p* < 0.001, and all other 5 years, *cov* = −0.01, 95 % CI = [−0.014, −0.002], *r* = −0.04 to −0.05, *p* = 0.01.

With respect to our research aim, adolescent SAD symptoms were significantly associated with less peer involvement one year later, *b* = −0.03, 95 % CI = [−0.064, −0.005], *β* = −0.04 to −0.05, *p* = 0.02. In turn, peer involvement was significantly associated with a lower probability of cannabis non-use 1 year later, *b* = −1.53, *OR* = 0.22, 95 % CI_*OR*_ = [0.144, 0.326], *p* < 0.001, and a significantly higher cannabis frequency 1 year later, *b* = 0.23, *IRR* = 1.25, 95 % CI_*IRR*_ = [1.012, 1.551], *p* = 0.04. Most importantly, there appeared to be significant indirect effects from adolescent SAD symptoms via adolescents’ peer involvement 1 year later to adolescent cannabis non-use 2 years later, *b* = 0.05, 95 % CI = [0.005, 0.100], *p* = 0.03, but not cannabis frequency, *b* = −0.01, 95 % CI = [−0.018, 0.002], *p* = 0.12. All aforementioned associations were found controlling for reverse paths from cannabis frequency to higher peer involvement 1 year later, *b* = 0.01, 95 % CI = [0.003, 0.013], *β* = 0.02, *p* = 0.001, and from peer involvement to lower SAD symptoms 1 year later, *b* = −0.07, 95 % CI = [−0.119, −0.025], *β* = −0.05 to −0.06, *p* = 0.003. All of the reported cross-lagged associations represent fairly small effects.

## Discussion

This six-year longitudinal community study aimed to examine associations between SAD symptoms and cannabis use in adolescents from the general population. Specifically, we focused on the potential role of adolescents’ involvement with peers in longitudinal associations between SAD symptoms and cannabis use from early to late adolescence. Our results suggest that adolescent SAD symptoms were associated with less peer involvement 1 year later and that less peer involvement was, in turn, associated with less cannabis use 1 year later (Fig. 1). Most importantly, significant indirect effects suggest that lower levels of peer involvement may partially explain negative associations between SAD symptoms and cannabis use throughout adolescence. These results are more in line with the buffer perspective than the self-medication hypothesis. In other words, our findings suggest a developmental process in which adolescent SAD symptoms appear to precede lower levels of peer involvement and, in turn, appear to be longitudinally associated with lower levels of cannabis use.

### Adolescent SAD symptoms, peer involvement, and cannabis use

In line with previous research, our descriptive results suggested an increase in both the use of cannabis and the frequency of cannabis use during adolescence. At the same time, however, higher levels of SAD symptoms were longitudinally associated with higher probabilities of cannabis non-use and lower frequencies of cannabis use throughout adolescence, partially via lower levels of peer involvement. These results suggest a process in which adolescent SAD symptoms appear to interfere with adolescents’ involvement in the peer context (suggesting more social isolation) and may thereby be associated with reduced cannabis use. Since general engagement in health risk behaviors, including first contact and experimentation with cannabis, typically takes place in the peer context, opportunities for these experiences may be more limited for adolescents with higher levels of SAD symptoms [7, 17, 46]. These results are in line with the hypothesis that the inhibitory or protective role of adolescent SAD symptoms on cannabis use may be related to the social impairments that go together with high levels of these symptoms, in addition to the fearful traits that characterize high levels of SAD symptoms [18].

Interestingly, these results were found while controlling for longitudinal reverse positive paths from cannabis use to peer involvement and negative paths from peer involvement to SAD symptoms. Another interesting point is that all longitudinal cross-lagged paths could be constrained to be time invariant, which suggests that these associations appear to be similar throughout adolescence. Moreover, SAD symptoms were indirectly associated with cannabis non-use over time, via peer involvement, but not significantly to adolescents’ frequency of use. These results suggest that adolescent SAD symptoms and involvement with peers may play a different role in different aspects of adolescents’ involvement with cannabis, with a potentially stronger role of these two factors in the occurrence of cannabis use rather than the frequency of use. We should, however, note that all the associations were in the expected direction and we cannot rule out that a lack of power might have influenced our results.

As our results are in line with the buffer perspective, this seems to suggest that as problematic as high levels of SAD symptoms clearly are [2–4], high levels of SAD symptoms are not “all bad” for adolescents from the general population, as they seem to be associated with less engagement in risk behaviors such as (early) cannabis use. Although this developmental outcome with respect to cannabis use may seem healthier for adolescents with higher levels of SAD symptoms, this is not necessarily the case. Risk behaviors, including health risk behaviors such as cannabis use, appear to be part of normal adolescent development. Many of these risk behaviors represent healthy exploration and experimentation, and engaging in these behaviors likely contains elements of positive peer interaction and social adjustment as well [17, 46, 47]. From this point of view, the non-use of cannabis by adolescents with higher levels of SAD symptoms may be the result of reduced normative adolescent exploration and experimentation as well as dysfunctional peer relationships or social isolation, which could thereby be an indication of maladjustment. The peer context provides adolescents with many new social experiences and peer relationships have been shown to be important for a wide range of healthy developmental outcomes. This is because peer relationships provide opportunities for adolescents to explore boundaries, to learn about themselves and the world outside of the family context, and to practice social skills necessary for the establishment and maintenance of relationships with age-similar peers. In addition, peer relationships are instrumental in adolescents’ acquisition of other behavioral and emotional competencies [28–32]. Hence, adolescents with high levels of SAD symptoms may miss out on peer-related experiences—including prosocial ones—that are important for both their present and future adjustment in many areas.

Furthermore, the inhibitory effects of high SAD symptoms are likely not limited to cannabis use only. These effects likely translate to many other important areas of functioning, as exploration and experimentation is normative in many other aspects of adolescent development, such as different aspects of identity development (e.g., interpersonal, academic, occupational) and development of self-related constructs (e.g., self-concept clarity). SAD symptoms may thus not only limit adolescents’ engagement in risk behaviors, but also in other developmentally appropriate behaviors. Therefore, even though our results may seem to suggest better developmental outcomes for adolescents with higher levels of SAD symptoms regarding cannabis use, this is not necessarily the case when considering developmentally appropriate tasks and challenges during adolescence. Our results thereby illustrate the complex interplay of individual and social risk and promoting factors that may lead to positive outcomes in one developmental domain and negative outcomes in another domain.

### Strengths, limitations, and future research

An important strength of the present study is its six-year prospective longitudinal design, covering early to late adolescence. Whereas most previous research has been cross-sectional, the longitudinal design in our study allowed us to examine developmental processes and developmental order between adolescent SAD symptoms and cannabis use throughout adolescence. Additionally, we focused on adolescent peer involvement as potentially underlying longitudinal associations between adolescent SAD symptoms and cannabis use. By focusing on two factors that are generally considered to be an individual risk (i.e., SAD symptoms) or a social facilitation (i.e., peer involvement) for adolescent development, our study suggests that these factors may interact in a complex manner; a risk factor may still be able to predict better outcomes, depending on the developmental domain. Finally, our ZIP model correctly dealt with the skewed and zero-inflated distribution of adolescent cannabis use as well as distinguished between different aspects of use (i.e., cannabis non-use and frequency of use).

Our results should, however, be considered in light of some limitations. First, cross-lagged panel modeling only allows for inferences about temporal associations and we cannot draw any causal conclusions from these models. Second, our study sample was characterized by adolescents from relatively high SES families and our results should be interpreted in light of this sample characteristic. Third, we exclusively relied on adolescent self-reports. A multi-informant approach, such as including parent or peer reports, could also provide important information regarding associations between adolescent SAD symptoms, peer involvement, and cannabis use. However, as adolescents appear to be better judges of their own anxiety symptoms than, for example, parents [48, 49], adolescents remain essential informants. Fourth, we used a rather global assessment of adolescents’ peer experiences by focusing on how much time adolescents’ spend with peers. Even though the availability of cannabis itself as well as exposure to peer influences and potential modeling of peer behavior likely increase when spending more time with peers, adolescent peer experiences include many other aspects that may be important to consider in association with both adolescent SAD symptoms and adolescent cannabis use (e.g., quality of peer relationships, peer norms, or type of peer context). Furthermore, reliability of our 5-item peer involvement measure was relatively modest over time (i.e., *α* = 0.66–0.72). This might suggest that time spend with peers on different days of the week or in different contexts may capture slightly different aspects of adolescent peer involvement that may be differentially associated with both adolescent SAD symptoms and adolescent cannabis use. Future research may want to conduct a more in-depth investigation of the role of different aspects of adolescents’ peer experiences in relation to SAD symptoms and cannabis use, as well as examine other potentially important processes underlying associations between these two constructs.

Fifth, considering the many studies providing support for risk processes between SAD symptoms and (problematic) cannabis use (i.e., self-medication hypothesis), the protective processes found in the present study may apply only to (*i*) adolescents, (*ii*) from the general population, (*iii*) including current users and non-users of cannabis as well as adolescents which have and have not initiated cannabis use yet, and (*iv*) with relatively low levels of SAD symptoms as well as cannabis use. High SAD symptoms in adolescents from the general population may act as a protective factor initially, but as development goes awry and SAD symptoms and/or cannabis use reach more problematic levels this may ‘activate’ some of the risk processes suggested by the self-medication hypothesis. Some have also suggested that processes in line with the self-medication hypothesis may not emerge until adulthood [50]. This argues for the importance of studying associations between factors throughout development, because certain factors (such as SAD symptoms) may act as a buffering factor in one developmental period but may constitute a risk factor in another period. In addition, certain developmental processes, such as the peer context during adolescence, may be more salient in one developmental period than another. Future research should aim to gain more insight into the exact nature of the relationship between anxiety and cannabis use in adolescents and young adults from the general population, especially regarding the specific circumstances under which risk or protective processes may link SAD symptoms to cannabis use. Such information would be highly salient to prevention and intervention practices.

Finally, even though Dutch policies regarding cannabis use might be considered fairly permissive in comparison to other countries, nationally representative numbers suggest that Dutch adolescents’ cannabis use is comparable to the United States and other European countries [12]. Also, our results are in line with studies from the United States and [24] and Finland [25] finding preliminary support for negative associations between SAD symptoms and cannabis use in adolescent community samples. Therefore, we believe that the results of this study are not necessarily limited to Dutch adolescents’ from the general population, but may apply to adolescents in the United States and other European countries as well.

## Conclusion

In research on associations between SAD symptoms and cannabis use there has been only limited attention to adolescents from the general population. Results from the present study describe a process in which adolescent SAD symptoms appear to limit peer involvement and, in turn, appear to be longitudinally associated with less cannabis use in the general population. This developmental process is in line with the buffer perspective that suggests that high levels of SAD symptoms may protect adolescents in the general population from (early) cannabis use. This study and other studies [24–26] suggest that results from clinical and adult samples may have been incorrectly assumed to generalize to adolescent community samples. As noted, we should, however, be cautious in interpreting this finding as a purely positive developmental outcome. Experimentation with (health) risk behaviors, including cannabis use, is a normal part of adolescent development and contains elements of positive peer interaction and social adjustment. The inhibitory effects of high levels of adolescent SAD symptoms are likely not limited to adolescents’ engagement in risky behaviors only, but also reduce developmentally appropriate adolescent behaviors. Overall, our results illustrate the complex interplay of risk and promoting factors within the individual and in the social environment, which may lead to positive outcomes in one developmental domain and negative outcomes in another domain.

## Footnotes

1 Additional results of longitudinal measurement invariance tests suggested that our measures of both SAD symptoms and peer involvement showed longitudinal invariance from early to late adolescence.

2 Maximum likelihood estimation with standard errors and Chi-square robust to non-normality (MLR) corrects standard errors of estimates. Bootstrapped effects are therefore not available in M*plus* in combination with MLR estimation [37]. We checked results in our final model using regular maximum likelihood estimation (ML) with bias-corrected bootstrapped confidence intervals [51], and results were comparable.

3 A full correlation matrix is available from the first author on request.

4 Using multi-group analyses, we also explored potential moderation by sex of the longitudinal cross-lagged paths in the final model. These results should however be interpreted with caution, as the cases/parameter ratio suggested that these complex models actually require a much larger sample size for reliable estimation ([45], p. 111). Results suggested no significant moderation of the cross-lagged paths, with the exception of peer involvement predicting a lower probability of cannabis non-use for girls, *b* = −2.12, *OR* = 0.12, *p* < 0.001, compared to boys, *b* = −1.22, *OR* = 0.30, *p* < 0.001 (Wald test = 4.31, *df* = 1, *p* = 0.04). This also implied stronger indirect effects from SAD symptoms, via peer involvement, to cannabis non-use for girls, *b* = 0.07, 95 % CI = [0.001, 0.136], *p* = 0.046, compared to boys, *b* = 0.04, 95 % CI = [0.001, 0.078], *p* = 0.047.
